# Salmon DNA Accelerates Bone Regeneration by Inducing Osteoblast Migration

**DOI:** 10.1371/journal.pone.0169522

**Published:** 2017-01-06

**Authors:** Ayako Sato, Hiroshi Kajiya, Nana Mori, Hironobu Sato, Tadao Fukushima, Hirofumi Kido, Jun Ohno

**Affiliations:** 1 Division of Oral Implantology, Department of Oral Rehabilitation, Fukuoka Dental College, Tamura, Sawara-ku, Fukuoka, Japan; 2 Research Center for Regenerative Medicine, Fukuoka Dental College, Tamura, Sawara-ku, Fukuoka, Japan; 3 Division of Cellular Physiology, Department of Physiological Science and Molecular Biology, Fukuoka Dental College, Tamura, Sawara-ku, Fukuoka, Japan; 4 Division of Periodontology, Department of Odontology, Fukuoka Dental College, Tamura, Sawara-ku, Fukuoka, Japan; 5 Division of Fixed Prosthodontics, Department of Oral Rehabilitation, Fukuoka Dental College, Tamura, Sawara-ku, Fukuoka, Japan; Medical University of South Carolina, UNITED STATES

## Abstract

The initial step of bone regeneration requires the migration of osteogenic cells to defective sites. Our previous studies suggest that a salmon DNA-based scaffold can promote the bone regeneration of calvarial defects in rats. We speculate that the salmon DNA may possess osteoinductive properties, including the homing of migrating osteogenic cells. In the present study, we investigated the influence of the salmon DNA on osteoblastic differentiation and induction of osteoblast migration using MG63 cells (human preosteoblasts) *in vitro*. Moreover, we analyzed the bone regeneration of a critical-sized *in vivo* calvarial bone defect (CSD) model in rats. The salmon DNA enhanced both mRNA and protein expression of the osteogenesis-related factors, runt-related transcription factor 2 (Runx2), alkaline phosphatase, and osterix (OSX) in the MG63 cells, compared with the cultivation using osteogenic induction medium alone. From the histochemical and immunohistochemical assays using frozen sections of the bone defects from animals that were implanted with DNA disks, many cells were found to express aldehyde dehydrogenase 1, one of the markers for mesenchymal stem cells. In addition, OSX was observed in the replaced connective tissue of the bone defects. These findings indicate that the DNA induced the migration and accumulation of osteogenic cells to the regenerative tissue. Furthermore, an *in vitro* transwell migration assay showed that the addition of DNA enhanced an induction of osteoblast migration, compared with the medium alone. The implantation of the DNA disks promoted bone regeneration in the CSD of rats, compared with that of collagen disks. These results indicate that the salmon DNA enhanced osteoblastic differentiation and induction of migration, resulting in the facilitation of bone regeneration.

## Introduction

Recent strategies for bone tissue engineering incorporate an interactive triad of viable osteocompetent cells, soluble osteoinductive factors, and osteoconductive scaffolds, with the aim of achieving satisfactory bone regeneration within the defects [1,2]. The use of optimal scaffolds as osteoconductive constructs, are required for the delivery of osteogenic cells from the host tissue to the replacement tissue in the bone defects. Within the replaced tissue, osteogenic cells differentiate and deposit new bone.

We recently developed a unique biomaterial comprised of a mixture of salmon DNA and protamine that can be used as a scaffold for tissue engineering or drug delivery systems [3,4]. Our group also reported that the control of cell viability, flowability, soft tissue response, and biodegradation rate was dependent on the molecular weight of the DNA within the DNA and protamine complexes [5,6]. These results suggest that DNA is a potent tissue engineering candidate for use in biomaterials. Furthermore, we demonstrated that the DNA and protamine complexes facilitated bone regeneration in rat calvarial defects [7,8]. In addition, the cells expanded from the DNA and protamine engrafted defects exhibited osteogenic potential [7]. However, the role of these complexes in the acceleration of osteogenesis remains undetermined.

DNA can exhibit the property of releasing phosphates that have a strong affinity for calcium ions. The binding of phosphates to calcium forms calcium phosphate, which is a large constituent of bone minerals. Previous studies provide evidence that extracellular phosphate promotes osteogenic differentiation and calcification in preosteoblasts [9,10] and mesenchymal stem cells [11,12]. These events led us to hypothesize that DNA could stimulate osteocompetent cells, recruit them to a bone healing site, and undergo osteogenic differentiation during the healing process.

The aim of this study is to elucidate whether the salmon DNA can induce the migration of osteogenic cells, osteoblastic differentiation, and bone regeneration. In the present study, we have examined the effects of salmon DNA on the migration and differentiation of MG63 cells (human pre-osteoblasts) *in vitro*, as well as bone regeneration of calvarial defects in an *in vivo* rat model.

## Materials and Methods

### Preparation of the DNA and DNA disk

Sterilized salmon testis DNA containing more than 20,000 bp DNA (Maruha-Nichiro Holdings Ltd., Tokyo, Japan) was used in this study. The DNA was mixed with distilled water to convert it into a jelly. The DNA jelly was injected into a silicone mold (internal diameter, 8 mm; height, 0.8 mm) on a polytetrafluoroethylene plate, and freeze-dried. Subsequently, the fabricated DNA disks (5 μg) were immediately and carefully removed from the polytetrafluoroethylene plate and silicone mold.

### Rat implantation model

This study was performed in strict accordance with the recommendations stated in the Guide for the Care and Use of Laboratory Animals of the National Institutes of Health. Animal studies were conducted in accordance with the protocols approved by the Animal Care and Use Committee of Fukuoka Dental College (No. 13009). We used a total of 80, 10-week-old male Sprague-Dawley (SD) rats (weight of approximately 300 g), purchased from KBT Oriental Co., LTD (Tosu, Japan). All surgeries were performed under general anesthesia induced 2% isoflurane (Abbott Laboratories, Abbort Park, IL, USA) and an air mixture gas machine (Anesthesia machine SF-B01; MR Technology, Inc., Tsukuba, Ibaraki, Japan). All efforts were made to minimize animal suffering.

To examine both the biocompatibility and biodegradability of the DNA disks, we performed a subcutaneous implantation of the disks [6]. Briefly, an incision was made in the backs of the rats and the fabricated disks were implanted subcutaneously. After the insertion of the samples, the soft tissues were closed in separate layers by suturing. The controls consisted of a group of sham-operated rats that did not undergo sample implantation. Samples from both groups [experimental (n = 5 at each time point) and control groups (n = 5 at each time point)] were obtained on days 1, 3, and 7 days after the implantation.

A critical-size calvarial bone defect (CSD) was created and treated with an 8-mm diameter paste disk for each of the different observation times [7,13]. After shaving the skin, an incision was made in the skull, and the periosteum was opened to expose the surface of the calvarial bones. A circular bone defect (full-thickness, 8 mm diameter) was created in the left parietal bone with a trephine drill and irrigated with saline to remove any bone debris. The DNA disks (n = 5 at each time point) were then implanted into the defects. Controls for the transplant experiments included calvarial defects implanted with (n = 5 at each time point) or without (n = 5 at each time point) a CollaPlug (Zimmer Biomet Holdings, Inc., Warsaw, USA), an absorbable collagen wound dressing to be used in dental surgery.

### Tissue preparation

The tissue samples were removed after euthanasia of the rats by injecting an overdose of isoflurane. Samples from the subcutaneous implantation tissues were removed on days 1, 3, and 7 days. The specimens were fixed in 4% paraformaldehyde in PBS and embedded in paraffin. The replaced fibrous connective tissues were removed from the CSD on day10. The specimens were immediately frozen in liquid nitrogen, and serial frozen sections (4 μm) were used for an immunohistochemistry. Rat calvaria from the experimental and control groups were removed at 1, 2, and 3 months. The specimens were fixed in 4% paraformaldehyde in PBS, decalcified in 10% Ethylenediamine tetraacetic acid (EDTA) for three weeks at 4°C and were then embedded in paraffin. The paraffin sections were stained with hematoxylin and eosin (H&E) to visualize any histological changes.

### Bone regeneration evaluations

Bone regeneration was evaluated using an in vivo micro-computed tomography (micro-CT) system (Skycan-1176 micro-CT; Bruker, Kontich, Belgium) at 50 kVp and 500 μA for rats while under anesthesia at 1, 2, and 3 months following the disk implantation. Each image data set consisted of a scan size of approximately 35 μm. The percentage of the newly formed bone in a calvarial bone defect (New-Bone %) was calculated as previously described [7,14].

### Cell culture

Human preosteoblast MG63 cells were cultured in Dulbecco’s modified Eagle’s medium (DMEM; Invitrogen, Tokyo, Japan) with 10% (v/v) fetal bovine serum (FBS; HyClone, Logan, UT, USA) and 1% (v/v) penicillin/streptomycin (PS; Invitrogen). All cultures were maintained at 37°C in a humidified incubator and with 5% CO_2_. Upon reaching confluence, the cells were cultured in DMEM with FBS supplemented with osteogenic induction medium (OIM) containing ascorbic acid, dexamethasone and β-glycerophosphate (TaKaRa, Tokyo, Japan). The cells were incubated in the OIM-containing culture medium with or without the salmon DNA fragment (100 μg/mL) for 0–21 days.

### Explant outgrowth culture of the replaced fibrous connective tissue from CSD

**Fig 1:**
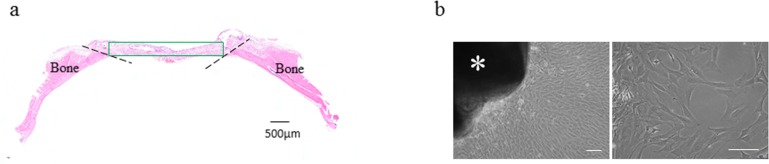
Tissue explants for outgrowth culture. (a) The replaced fibrous connective tissue (rectangle) in the bone defects at 10 days was harvested and used for the explant outgrowth culture system. The dotted lines indicate the edge of the host bone. Scale bar = 500 μm. (b) Phase contrast images show the growth initiation of fibrous connective tissue in the bone defects under the explant outgrowth culture. Spindle-shaped cells were outgrown from the explant (left). Morphological characteristics are indicated at a high magnification (right). * indicates the explant. Scale bars, 100 μm.

Fibrous connective tissue from the calvarial defects on day 10 after DNA disk implantation was used for an explant outgrowth culture system. The primary culture of the mesenchymal cells was obtained as outgrowths from the explants of the fibrous connective tissue from the calvarial defects (Fig 1A). The tissues were cut into pieces of approximately 3 × 2 × 2 mm and were placed on the bottom of 60-mm dishes. They were allowed to adhere to these dishes and then cultured in DMEM supplemented with 10% FBS (v/v) and 1% (v/v) penicillin/streptomycin. All cultures were maintained at 37°C in a humidified incubator and with 5% CO_2_. After 14 days, the outgrown cells (Fig 1B) from the explants were subcultured until confluent and then transferred into the medium in the presence or absence of OIM for seven days. These cells were then used for subsequent immunocytochemical examination.

### Cell proliferation assay

MG63 cells were seeded into 96-well plates with 100 μL of medium at a density of 1x10^4^ cells, and incubated at 37°C overnight to allow the adherent cells to attach to the walls. The cultured cells were then incubated with or without DNA (50–150 μg/mL) for 0, 1, 3, 5, or 7days. Next, 10 μL of cell counting kit-8 solution (CCK-8, Dojindo Laboratories, Kumamoto, Japan) was added to each well and the cultured cells were incubated at 37°C for 4h. After incubation, the amount of formazan dye generated by cellular dehydrogenase activity was measured by absorbance at 480 nm as the detection wavelength and at 650 nm as the reference wavelength using a plate reader (Multiskan JX, Labsystems Oy, Helsinki, Finland). Cell proliferation ratio was expressed as a percentage relative to untreated cells.

### Real-time reverse transcription-polymerase chain reaction (qRT-PCR)

The total RNA was extracted from the harvested cells using the ISOGEN reagent. First-strand cDNA was synthesized from the total RNA (5 μg) using SuperScript II reverse transcriptase, according to the manufacturer’s instructions. The resulting templates were amplified using an Applied Biosystems 7500 Real-Time PCR System (Life Technologies, Carlsbad, CA, USA). Nucleotides, TaqDNA polymerase, and a buffer were included in the SYBR Premix Ex Taq II (TaKaRa, Tokyo, Japan). Glyceraldehyde-3-phosphate dehydrogenase (*GAPDH*) was used as an internal control. The relative mRNA expression was normalized as the ratio of runt-related transcription factor 2 (*Runx2)*, alkaline phosphatase (*ALP*) or osterix (*OSX*) mRNA to the level of *GAPDH* expression levels. All of the reactions were run in hexaplicate. The primers used were as follows: *GAPDH*, forward primer 5’-AGCCACATCGCTCAGACAC-3’ and reverse primer, 5’-GCCCAATACGACCAAATCC-3’; *Runx2*, forward primer 5’-TGGACGAGGCAAGAGTTTCACC-3’ and reverse primer, 5’-CTTCTGTCTGTGCCTTCTGGGTTC-3’; *ALP*, forward primer 5’-CCTGCCTTACTAACTTAGTGC and reverse primer, 5’-CGTTGGTGTTGAGCTTCTGA-3’; *OSX*, forward primer 5’-CATCTGCCTGGCTCCTTG-3’ and reverse primer, 5’-CAGGGGACTGGAGCCATA-3’.

### Western blot analysis

The cells were lysed in Cell Lysis Buffer (Cell Signaling Technology, Delaware, CA, USA) containing a 1x Protease/Phosphatase Inhibitor Cocktail (Cell Signaling Technology). The protein content was measured with a protein assay kit (Pierce, Hercules, CA, USA). Protein samples (15 μg), together with a protein marker (Precision Plus Protein Western C Standards; Bio-Rad), were separated on 12% Mini-Protean TGX gels (Bio-Rad, Richmond, CA, USA) for 30 min at 200 V. The separated gels were transferred to a polyvinylidene fluoride (PVDF) membrane for 3 min using the Trans-Blot Turbo Transfer system (Bio-Rad) with Trans-Blot Transfer Packs. Western blots with Runx2), ALP, OSX, and β-actin (ACTB) were processed on the iBind Western System (Life Technologies, Carlsbad, CA, USA) as specified by the antibody manufacturer (anti- Runx2) [Abcam, Tokyo, Japan], anti-ALP [Abcam], anti-Sp7/osterix [OSX; Abcam], and anti- ACTB [Bio-Rad] primary antibodies; and horseradish peroxidase-conjugated anti-mouse secondary antibody [Bio-Rad]). The incubated membranes were developed using an enhanced chemiluminescence system (SignalFire Plus ECL Reagent; Cell Signaling Technology). The band density was quantified using the NIH-Image J software and normalized to that of ACTB on day 0.

### Immunofluorescence

Cultured cells on 10-well glass slides were fixed with 4% paraformaldehyde for 10 min and washed in 0.1% TritonX-100 in PBS for 15 min. The cells were incubated with anti-RUNX2 (1:100; Abcam), anti-osteopontin (OPN, 1: 500; Abcam), anti-alkaline phosphatase (ALP, 1:100; Abcam), or anti-Sp7/osterix (OSX, 1:100; Abcam), at 4°C overnight. After washing with PBS, the cells were incubated with anti-rabbit IgG or anti-mouse IgG conjugated with Alexa Fluor 488 or 568 (1:200; Molecular Probes, Eugene, OR, USA) at room temperature for 45 min. To visualize the nuclei, cells were counterstained with 4, 6-diamidino-2-phenylindole (DAPI; Vector Laboratories, Inc., Burlingame, CA, USA).

Frozen sections of the replaced connective tissue from CSD were used for the detection of aldehyde dehydrogenase 1 (ALDH1) activity and OSX expression. For the detection of ALDH1 activity in the frozen sections, we used an Aldefluor kit (Stemcell Technology, Vancouver, Canada), according to the manufacturer’s instructions. To examine OSX expression, we performed an indirect immunofluorescence method. Briefly, the frozen sections were fixed in cold acetone for 10 min and then incubated with anti-Sp7/osterix (OSX, 1:100; Abcam), at 4°C overnight. After washing with PBS, they were incubated with anti-mouse IgG conjugated to Alexa Fluor 488 (1:200; Molecular Probe) for 45 min at room temperature. DAPI was used for the nuclear staining.

### ALP staining

MG63 cells were stained with an ALP kit (Sigma-Aldrich, St. Louis, USA), according to the manufacturer’s instructions, after an incubation for the indicated period with OIM in the presence or absence of salmon DNA fragments.

### Transwell migration assay

The effects of DNA on MG63 cell migration were evaluated using a transwell insert system. MG63 cells were seeded at a density of 5 × 10^4^ cells/well onto 8 μm transwell inserts. The lower chamber was filled with 800 μL DMEM with 10% FBS alone or medium containing DNA (100 μg/mL). The wells of the upper chamber received MG63 cells. Medium without adding DNA was used as negative control. The cells were incubated for 24, 48, and 72 h at 37°C and then stained with DAPI. Migration activity was evaluated as the percent migration of cells from the upper chamber of the transwell insert to the lower chamber in the three high power fields (× 100) per well. MG 63 cell migration toward the DNA was examined via a transwell migration assay. The experiment was performed in triplicate.

### Statistical analysis

A statistical analysis used SPSS (IBM^®^ SPSS Statistics, version 19). The analysis was performed with one-way analysis of variance (ANOVA), and Bonferroni’s multiple comparison test to determine the statistical differences among the samples. Data are presented as the mean ± standard deviation (SD) and p values < 0.05 were considered to be statistically significant.

## Results

### Effects of DNA on cell proliferation and biodegradability

**Fig 2:**
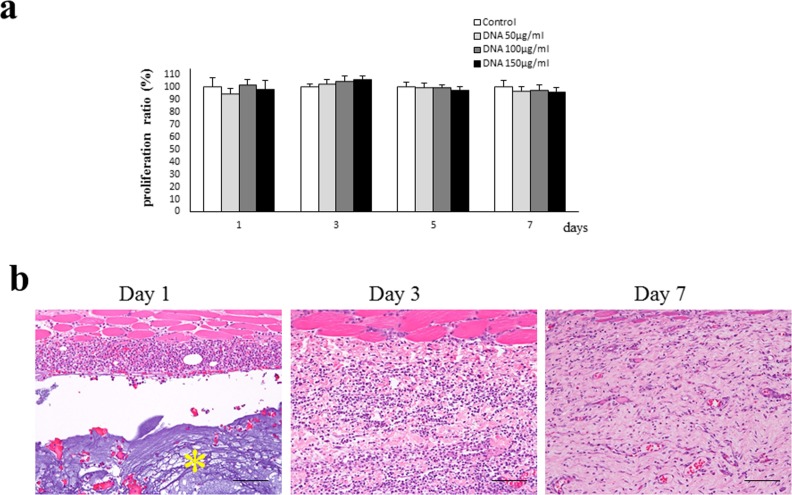
Effects of DNA on cellular proliferation and in vivo biodegradability. (a) A CCK-8 cell proliferation assay was conducted on MG63 cells treated with or without DNA at different time points. The results are expressed as the proliferation ratio (%) relative to control. The data shown represent the mean±standard deviation of five independent experiments, each performed in triplicate. There were no significant differences (p > 0.05) between any of the comparisons. (b) Representative histologic images show the biodegradability of an implanted DNA disk into the subcutaneous tissue of the skin in rats on days 1, 3, and 7. Paraffin sections were stained with a hematoxylin and eosin solution. * indicates the implanted disk. Scale bars, 50 μm.

To examine the effect of DNA on cell proliferation, we conducted a CCK-8 assay on MG63 cells treated with or without DNA (50, 100, and 150 μg/mL). Fig 2A shows no inhibitory effects on the DNA-treated cells, compared with the control. There were also no significant differences (p > 0.05) in the cellular proliferation between the different samples at 50 μg/mL, 100μg/mL, and 150 μg/mL. These results indicate that DNA treatment exhibits no cytotoxic effects on the cultured cells.

Next we histologically evaluated the biocompatibility and biodegradability of the DNA disks transplanted into the subcutaneous tissue of skin in rats (Fig 2B). The implanted DNA disk was surrounded by a band-like infiltrate of neutrophils on day 1 after the implantation. No bacterial colonies were observed. On day 3, the implanted disk was completely degraded and replaced by inflammatory granulation tissue containing many lymphocytes and several plasma cells. No foreign-body giant cells were observed in the granulation tissue. The cellular granulation tissue in the implant sites was transferred to the fibrous connective tissue containing a few inflammatory cells and vascular slits on day 7. These results indicate that the biodegradation of the DNA disks is complete before day 3 after the implantation and suggest that a giant cell-independent foreign-body reaction contributes to the biodegradation.

### DNA accelerates osteogenesis in MG63 cells

**Fig 3:**
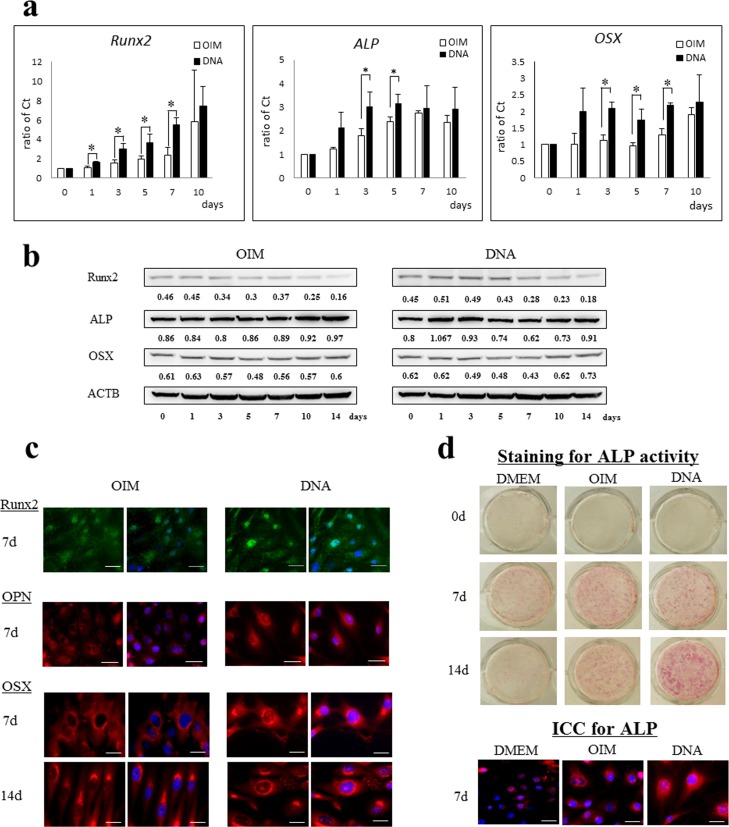
Promotion of osteogenesis in MG63 cells by the addition of DNA. (a) mRNA expression of the osteogenesis markers,*Runx2*, *ALP*, and *OSX*, by qRT-PCR methods. The results are expressed as the mRNA fold-increases (normalized to GAPDH mRNA) and compared with the results for the MG63 cells treated with OIM on day 0. The data shown represent the mean±standard deviation from five culture wells, each performed in triplicate. *, significantly different at p<0.05 compared with the cells cultured with OIM-medium alone. (b) Protein expression of Runx2, ALP, OSX, and ACTB, in MG63 cells treated with or without DNA, by a Western blot. Quantification of proteins was performed using the NIH Image J software and values shown in the part were the fold increase normalized to those of ACTB on day 0. Similar results were obtained in five independent experiments. (c) Immunofluorescence of Runx2 (green), OPN (red), and OSX (red) in the cells treated with or without DNA on days 7 and 14. 4’,6-diamidino- 2-phenylindole (DAPI) was used to label the nuclei (blue). Scale bars, 25 μm. (d) Staining for ALP activity and protein (ALP, red; DAPI, blue) in the cells incubated with or without DNA on days 0, 7, and 14. Scale bars, 25 μm. Runx2, runt-related transcription factor-2; ALP, alkaline phosphatase; OPN, osteopontin; OSX, osterix; ACTB, β-actin; ICC, immunocytochemistry.

To investigate whether DNA-treatment can promote osteogenesis in MG63 cells cultured in the presence of OIM, we first analyzed the gene expression of osteogenesis markers in the OIM-exposed cells using qRT-PCR method. Fig 3A shows the mRNA expression of *Runx2*, *ALP*, and *OSX* in the MG63 cells cultured with OIM containing DNA compared with the OIM alone. mRNA expression of the osteogenesis-related genes was significantly increased in MG cells cultured with OIM containing DNA. The relative *Runx2* mRNA expression in the MG63 cells treated with DNA gradually increased by day 10. In contrast, the enhanced mRNA expression of *ALP* and *OSX* reached a plateau on days 3 and 1, respectively.

We next examined whether the expression of osteogenic proteins, such as Runx2, ALP, and OSX, was upregulated in MG63 cells cultured with OIM containing DNA (Fig 3B). Runx2 expression was upregulated in cells exposed to the DNA at early time points (days 1–5). An upregulation of ALP expression was observed in the cells treated with DNA on days 1 and 3, while ALP expression in the cells exposed to OIM alone increased after day 10. OSX expression was also upregulated in the cells cultured with DNA, compared with those exposed to OIM alone.

Next, we performed an immunocytochemical method to detect the expression of Runx2, OPN, and OSX expression in MG63 cells treated with or without DNA (Fig 3C). Runx2 expression was observed within the nuclear portion of both the cells treated with or without DNA on day 7. However, the intensity of the nuclear expression was substantially increased in the cells exposed to DNA compared to those treated with OIM alone. Similarly, intensely perinuclear expression of OPN was observed in the cells treated with DNA on day 7, compared with those without DNA. MG63 cells treated with DNA demonstrated juxtanuclear and intranuclear accumulation of an immunofluorescent reaction with OSX on day 7. The intensity of both the juxta- and intranuclear expression of OSX was increased in the DNA-treated cells on day 14. In contrast, MG63 cells treated with OIM alone showed perinuclear expression of OSX on day 7. Although an intense juxtanuclear expression was apparent on day 14; the intranuclear portion remained unreactive. These immunocytochemical results indicate that transcriptional proteins for osteoblastic differentiation were prepared by the MG63 cells treated with DNA by day 7.

We finally examined the ALP staining intensity in the cells treated with or without DNA because the histochemical detection of ALP activity is considered to be an early marker of osteogenesis [11,15]. The intensity of ALP staining was significantly enhanced in DNA-treated cells on day 14, compared with the cells cultured with or without OIM alone (Fig 3D). Immunocytochemical findings of ALP protein expression corresponded with the intensity of ALP activity staining. Intensely cytoplasmic expression of ALP protein was observed in DNA-treated cells on day 7 (Fig 3D). The addition of DNA to the OIM culture appeared to stimulate an earlier onset of osteogenic differentiation in the MG63 cells based on mRNA and protein expression of osteogenic factors and ALP activity.

### DNA induces osteogenic cell migration

**Fig 4:**
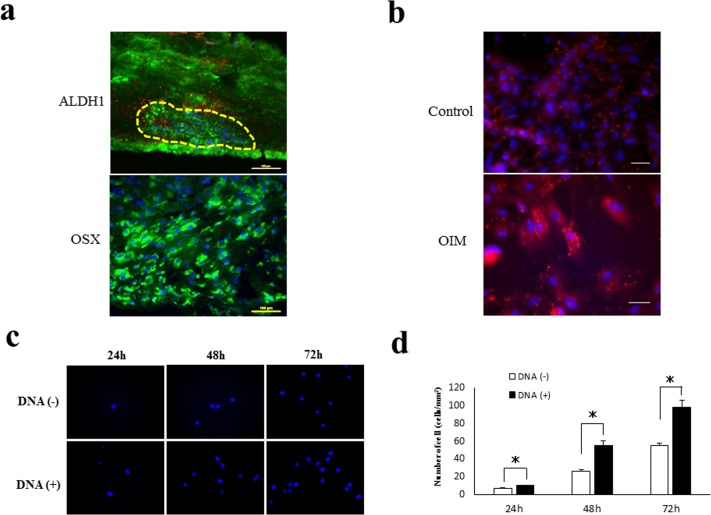
Migration inducer of DNA to osteogenic cells. (a) Fluorescence images of ALDH1 activity and OSX expression in the replaced fibrous connective tissue on the frozen sections of the replaced tissue in the bone defect. Accumulation of ALDH1-positive cells in the dotted circle. Scale bars, 100 μm. (b) Immunocytochemical detection of OSX in outgrown cells, taken from the connective tissue of the bone defect, cultured with or without OIM. Scale bars, 100 μm. (c) Representative fluorescent pictures of migrated MG 63 cells toward the DNA using a transwell migration assay. The migrated cells were stained with DAPI (blue). (d) The figure represents the results of three different experiments expressed as the mean±standard deviation. *, significantly different at p < 0.05 compared with the cells cultured without DNA.

The initial step in the regeneration and repair process is the replacement of bone defect by the fibrous connective tissue. The replaced connective tissue may consist of numerous osteogenic cells, such as mesenchymal stem cells and osteoblastic progenitor cells. Osteogenesis-inducible materials are necessary for the induction of osteogenic cell migration to the regenerative sites. To investigate the effect of DNA on the migration of osteogenic cells, we first demonstrated the histochemical and immunohistochemical detection of ALDH1 and OSX, respectively. This was performed using frozen sections of replaced connective tissue from calvarial bone defects in the rats on day 10 following the transplantation of the DNA disk. The histochemical activity of ALDH1 is known to be one of the functional markers for mesenchymal stem cells (MSC) [16,17]. We applied the Aldefluor assay [18,19] to the frozen sections containing the bone defects. Cells positive for ALDH1 activity tended to accumulate in a scattered pattern within the replaced fibrous connective tissue (Fig 4A). In contrast, the OSX-positive cells were observed diffusely in the replaced connective tissue (Fig 4A). These findings indicate that the replaced connective tissue contained both MSC-like and osteoblast-like cells on day 10.

We also investigated the immunocytochemical detection of OSX in cells from an explant outgrowth culture system, to determine whether the cells obtained from the replaced connective tissue of the bone defect could retain an osteogenic-potential in themselves. OSX expression was observed in both the outgrown cells cultured with or without OIM for 7 days, indicating that the outgrown cells derived from the replaced connective tissue of the defect retain their osteogenic-potential (Fig 4B).

To elucidate whether DNA possesses the potential to induce osteogenic cells, we next performed a transwell migration assay. In these experiments, medium containing DNA (100 μg/mL) or medium alone was placed in the lower chamber and migration was initiated by the addition of the MG63 cells. Migration was allowed to proceed for 24, 46, and 72 h at 37°C, at which time the number of DAPI-positive cells (Fig 4C) that had adhered to the underside of the DNA-coated filter was determined. The number of cells that had migrated towards the DNA was increased by 1.6-fold [DNA(–) 6.5 ± 0.7 vs DNA(+) 9.9 ± 0.6; p = 0.03], 2.1-fold [DNA(–) 26.0 ± 1.7 vs DNA(+) 55.0 ± 5.2; p = 0.01], and 1.8-fold [DNA(–) 55.1 ± 2.3 vs DNA(+) 98.0 ± 8.3; p = 0.01] at 24, 48, and 72 h, respectively, compared with those in the control wells (Fig 4D). The data from the migration assay indicate that DNA can induce the migration of osteoblasts.

### Implantation of a DNA disk facilitates the regeneration of calvarial defects

**Fig 5:**
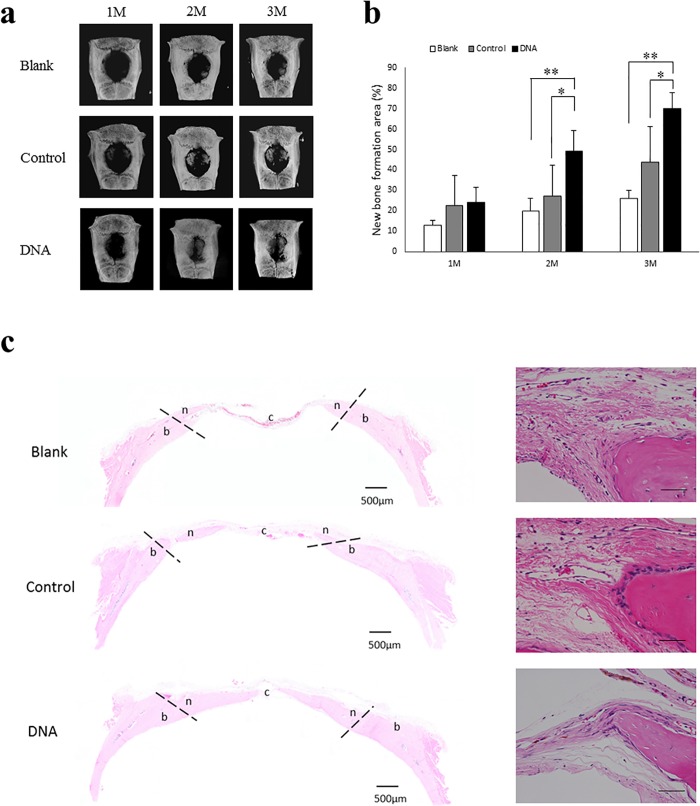
Acceleration of bone regeneration in DNA disk-implanted bony defects. (a) Calvarial healing assessed by micro-CT analysis at different time points. Procedures for calvarial defects included no treatment (Blank), Colla Plug-implant (Control), and DNA disk-implant (DNA). (b) Quantitative results for the micro-CT analysis. Healing of the defects is shown as a fraction of the new bone on the total defect area as quantified by the micro-CT analysis (n = 5 / group). The columns expressed as the mean±standard deviation.*, significantly different at p < 0.05 compared with control. **, significantly different at p < 0.01 compared with blank. Blank, untreated rats; Control, Colla Plug-implanted rats; DNA, DNA disk-implanted rats. (c) Panoramic images of decalcified, H&E-stained sections of the bone defects, no treatment (Blank), Colla Plug (Control)—and DNA disk (DNA)-implants in the rats at 3 months. Dotted lines indicate the edge of the host bone and the defect filled with the fibrous connective tissue, respectively. c, connective tissue; n, new bone; b, existing calvarial bone. Scale bars, 500μm. Right columns show a higher magnification of the newly formed bone. Scale bars, 100μm.

To investigate whether DNA can induce the potential for bone regeneration, we performed the implantation of a DNA disk, Colla Plug (control), or nothing (blank) into the CDS within the calvarial bones of 10-week-old SD rats. These CDS cannot spontaneously heal during the bone healing period [20]. We first evaluated the area of bone regeneration by micro-CT scanning. By 1-month postoperatively, the micro-CT scans revealed small peninsulas of new bone in the rats receiving both DNA and Colla Plug, while no evidence of new bone formation was observed in the untreated rats (Fig 5A, left columns). A peninsula extension showed an increase in the DNA disk implantation at 2 months, compared with control and blank implantations (Fig 5A, middle columns). Furthermore, there was substantially enhanced bone regeneration obtained from animals implanted with the DNA disk, compared with the other groups (Fig 5A, right columns).

We next quantified these micro-CT images to determine the percentage of defect healing by quantifying the pixels in these defects. The percentage of healing was determined by dividing the defect area by the defect size immediately following the operation (Fig 5B). There were no significant differences between any of comparisons [blank (white bars) 13.0 ± 2.4%; control (CollaPlug, gray bar) 22.5 ± 14.9%; DNA (black bar) 24.3 ± 7.1%]. Compared with the blank (19.8 ± 6.1%) and control (27.2 ± 15.3%) rats, DNA disk-implanted rats (49.1 ± 10.0%) consistently exhibited a higher percentage of new bone area after 2 months [Control vs Blank, p = 0.95; DNA vs Blank, p = 0.027; DNA vs Control, p = 0.004]. In both untreated (25.9 ± 4.2%) and collagen-implanted (44.0 ± 17.3%) rats, the defects had healed by less than 25% and 50%, respectively, over the course of 3 months. In contrast, the defects in the rats implanted with the DNA disk (70.2 ± 7.4%) showed greater than 70% healing after 3 months [Control vs Blank, p = 0.072; DNA vs Blank, p = 0.010; DNA vs Control, p = 0.000].

The histological analysis of the H&E-stained sections at 3 months was in agreement with the evidence of new bone regeneration in samples from the DNA disk-implantation by micro-CT findings (Fig 5C, left columns). Moreover, active osteoblast-like cells lined by the periphery of the newly formed bone in the CDS implanted with the DNA and Colla Plug (Fig 5C, right columns). These findings demonstrate that the DNA disk could facilitate bone regeneration in rat calvarial CDS.

## Discussion

A potential scaffold for bone regeneration is required for the migration of osteogenic cells (e.g. preosteoblasts and mesenchymal stem cells) from the surrounding tissues and the formation of a mineralized tissue. However, a scaffold implanted on the defects must achieve a desirable biocompatibility and biodegradation. After the process of biodegradation, the defects are replaced by fibrous connective tissue containing numerous osteogenic cells. In this study, we propose that DNA may represent a promising candidate material to make scaffold constructs for bone tissue engineering, due to its tissue compatibility and degradation, migration potential for osteogenic cells, as well as bone regeneration activity.

Evidence that DNA exhibits a desirable tissue compatibility and degradation was revealed in experiments of DNA disk implantation in the subcutaneous tissue of rats. The biodegradation process of the implanted materials is divided into two steps: 1) the humoral phase (absorption phase) is the initial step of biodegradation and is mediated by extracellular enzymes from both the blood plasma and cellular production; 2) the second step is a cellular phase, which is activated by phagocytes and foreign-body giant cells. Histological findings indicate that the degradation process of the implanted disk is not necessary for phagocytic activity. From these results, we suggest that deoxyribonuclease (DNase) in blood plasma [21,22] contributes to the degradation of the DNA disk in the subcutaneous tissue of the skin. Susceptibility to the biodegradation of the DNA disk may induce a satisfactory replacement of the bone defect by fibrous connective tissue containing osteogenic cells.

It has been reported that extracellular phosphate promotes osteogenic differentiation and calcification in pre-osteoblasts [9,10] and mesenchymal stem cells [11,12]. Moreover, our group recently reported a direct effect of released phosphates from the salmon DNA on the osteogenesis of cultured cells [23]. In our *in vitro* experiments, we found that the addition of DNA to the MG63 cell culture upregulated mRNA and protein expression of osteogenic-related factors, such as Runx2, OSX, and OPN. In vitro and in vivo studies reveal that both Runx2 and OSX are an essential transcription factor for osteoblast differentiation, matrix production, and mineralization [24]. OPN is expressed during the early stage of osteogenesis and is massproduction when calcification begins [25]. Similarly, ALP activity was upregulated in the MG63 cells cultured with OIM containing DNA. These results revealed that the salmon DNA released phosphates and increased the phosphate concentration in the culture medium. The released phosphates from the DNA provide a unique microenvironment that induces the acceleration of both the early and late differentiation of MG 63 cells. In particular, the transcellular uptake of phosphates is necessary for the production of matrix protein required for bone formation of the osteoblasts.

We found that the expression of osteogenesis-related proteins was observed in both the replaced tissues and the outgrown cells from the replaced fibrous connective tissue of the bone defects following DNA-disk transplantation. These results indicate that osteogenic cells containing MSCs migrate and accumulate in the replaced connective tissue of the defect following DNA-disk transplantation. In addition, our transwell migration assay demonstrated that the addition of DNA to the medium facilitated the migration potential of the MG63 cells. Taken together, these findings suggest that DNA can induce to the migration of osteogenic cells. The phosphates released from DNA may be involved in the acceleration of the osteogenic cell migration, although the precious mechanisms remained undetermined. A recent report demonstrated that the *in vitro* pathological levels of inorganic phosphates induced the cellular migration rate and calcification of human primary vascular smooth muscle cells [26]. In our study, the transcellular uptake of the released phosphates may play a crucial role in the acceleration of osteogenic cell migration and bone formation.

Our *in vivo* results are consistent with the data supporting that the addition of DNA can enhance the osteogenic differentiation of cultured cells. The micro-CT analysis of the DNA disk engrafted bone defects showed that the disk could facilitate efficient bone regeneration compared to the collagen-based constructs. Moreover, our histological findings confirmed that the bone defects following the DNA disk transplantation were replaced by an extension of new bone from the defect edges. We suggest that the DNA disk induces the proper healing of the bone defects because the new bone extension from the bone defect edges is believed to be a typical of bone healing. These results are supported by our previous studies demonstrating that the acceleration of bone regeneration was observed in the bone defects by the implanted DNA-based constructs [7,13,23].

In conclusion, the findings of this study will shed additional light on the role of DNA as a unique and promising candidate for osteogenic biomaterials. DNA exhibits favorable tissue compatibility and biodegradability, and facilitates the induction of osteogenic cell migration and osteogenesis. Furthermore, the DNA disk was found to accelerate bone regeneration in the rat calvarial defects. However, the detailed mechanisms, particularly regarding the release of phosphates, remains undetermined. Future studies should address the detailed mechanisms of DNA on the induction of osteogenesis in these bone defects.
